# Real-word adrenocorticotropic hormone treatment for childhood-onset nephrotic syndrome

**DOI:** 10.3389/fped.2023.1044075

**Published:** 2023-03-09

**Authors:** Ying Wang, Xiqiang Dang, Xiaochuan Wu, Yongzhen Li, Qingnan He, Xiaoyan Li

**Affiliations:** ^1^ Department of Pediatrics, The Second Xiangya Hospital, Central South University, Changsha, China; ^2^ Department of Pediatrics Nephrology, Children's Medical Center, The Second Xiangya Hospital, Central South University, Changsha, China; ^3^ Department of Pediatrics, The Third Xiangya Hospital, Central South University, Changsha, China

**Keywords:** nephrotic syndrome, adrenocorticotropic hormone, children, prednisone maintenance dose, relapse

## Abstract

**Background:**

Current first-line anti-proteinuric treatments do not produce a satisfactory therapeutic effect in a considerable number of patients with nephrotic syndrome (NS). Interest in adrenocorticotropic hormone (ACTH) for the treatment of NS has recently been revived. The present study investigated the efficacy and safety of ACTH treatment in children with frequent relapsing NS (FRNS), steroid-dependent NS (SDNS), and steroid-resistant NS (SRNS).

**Method:**

The ACTH treatment group was comprised of NS patients receiving ACTH treatment. Patients with serum cortisol concentrations <85.3 nmol/L and who had not received ACTH treatment previously were enrolled in the control group from January 2018 to January 2021. The maintenance dose of prednisone, the number of disease recurrences, the time of first disease relapse, immunosuppressant use, serum cortisol levels, and adverse events were recorded in both groups.

**Results:**

Fifty-one patients were included in the ACTH group, and twenty-one patients were enrolled in the control group. Concurrent treatment with one or more immunosuppressive and/or cytotoxic treatments occurred in 92.2% and 85.7% of patients in the ACTH and control groups, respectively, throughout the study period. A greater reduction in the prednisone maintenance dose was observed in the ACTH group compared with the control group after 1 year of follow up (0.603 ± 0.445 mg/kg vs. 0.267 ± 0.500 mg/kg, *p* = 0.006). During the one-year study period, fewer participants experienced one or more disease relapses in the ACTH group (45.1%) compared to the control group (76.2%, odds ratio = 3.896, *p* = 0.016). The number of disease recurrences per patient in the ACTH group was less than that in the control group (median difference = −1, *p* = 0.006). The mean length of remission was 8.902 m and 7.905 m in the ACTH group and control group, respectively. A log-rank test showed a longer relapse free survival for patients in the ACTH group (*p* = 0.046), but the Breslow test showed no significant difference between groups (*p* = 0.104). Ten patients in the ACTH group successfully discontinued all drug therapies. No patients in the control group were able to discontinue drug therapy as of February 2022.

**Conclusion:**

ACTH, combined with multiple drugs, is effective at reducing the prednisone maintenance dose and may effectively prevent disease relapses in childhood NS.

## 1. Introduction

Idiopathic nephrotic syndrome (NS) is one of the most common glomerular diseases in children, with an incidence of 1–3 per 100,000 children below 16 years of age (1–3). NS is characterized by heavy proteinuria, hypoalbuminemia, edema, and hyperlipidemia. Prolonged use of glucocorticoids continues to be the mainstay in the management of NS. Although approximately 85% of cases respond to corticosteroids (2, 4), most of these cases experience disease relapse and about half relapse frequently or become dependent on corticosteroids. These cases are termed frequent relapsing NS (FRNS) and steroid-dependent NS (SDNS), respectively (5). A small number of NS cases fail to achieve remission with corticosteroid treatment and are diagnosed as steroid-resistant NS (SRNS). The extensive side effect profile of glucocorticoids limits their use in prolonged therapy. A fraction of patients still relapse frequently or are unable to reduce corticosteroid use despite treatment with immunosuppressive medications. There remains an urgent need for effective, tolerable treatments for patients who frequently relapse, who do not respond to first- and second-line NS treatments, typically corticosteroids, cyclophosphamide, calcineurin inhibitors (CNIs), mycophenolate mofetil (MMF), and rituximab, or who are unable to tolerate first- and second-line NS treatments. Effective treatment for patients with FRNS, SDNS, or SRND is an ongoing challenge in clinical practice.

Adrenocorticotropic hormone (ACTH) is a pituitary polypeptide hormone that stimulates the adrenal glands to generate cortisol. ACTH was widely used to treat childhood nephrosis in the United States in the 1950s and was shown to be effective in ameliorating proteinuria and in improving patient survival (6). These effects of ACTH depended on the dose and duration of ACTH treatment. However, by the late 1960s, ACTH had largely been replaced by synthetic oral glucocorticoids due to the ease of glucocorticoids administration and the belief that ACTH acted by stimulating the production of corticosteroids (7). Recently, a number of case series of patients with steroid- and multidrug-resistant NS reported that ACTH effectively induces and maintains disease remission and improves glomerular filtration rate (GFR), suggesting that ACTH exerts steroidogenic-independent, proteinuria-reducing activity (8–10). The clinical responsiveness to ACTH therapy varies. ACTH has been ineffective at preventing disease relapses in a number of NS patients, and ACTH resistance has also been observed (5, 11, 12). However, several studies have found that a combination therapy with ACTH plus tacrolimus or CTX is well tolerated by patients with treatment resistant MGN, FSGS, and IgA nephropathy, and that this combination therapy significantly reduces proteinuria and improves clinical response rates compared with ACTH alone (13, 14).

Therefore, there is renewed interest in ACTH for the treatment of NS. Analysis of early clinical studies indicates an important need in expanding clinical experience and understanding ACTH treatment in NS. So far, most case series have been small and have only included adult experiences. To investigate the efficacy and safety of ACTH treatment in childhood patients with NS who have failed multiple previous therapies, the largest retrospective comparative study between two populations that received ACTH treatment or not to date was conducted. Relapse rate, glucocorticoid hormone reduction, and adverse events (AEs) in NS patients treated with ACTH within clinical practices were evaluated.

## 2. Materials and methods

### 2.1. Study design and patient population

A single-center, retrospective study was conducted to investigate the efficacy and safety of ACTH treatment for NS. The study was accepted by the Medical Ethics Committee of The Second Xiangya Hospital of Central South University (approval no. 2022–K035). The requirement for informed, written consent was waived, as the retrospective analysis was limited to preexisting data from medical records and collected by attending clinicians as a part of the patient's routine treatment. Data extracted from electronic medical records were coded with numbers to ensure confidentiality. The enrollment criteria of this study were as follows: (1) between 2 and 18 years old; (2) diagnosed SDNS/FRNS/SRNS. SDNS was defined as two consecutive disease relapses during therapy with prednisone or prednisolone (either at full dose or during tapering) or within 15 days of prednisone or prednisolone discontinuation. FRNS was defined as ≥2 relapses per 6 m within 6 m of disease onset or ≥4 relapses per 12 m within 12 consecutive months. SRNS was defined as persistent proteinuria after 4 weeks of therapy with daily prednisone or prednisolone at a standard dose (15); (3) serum cortisol assays had been performed for enrolled patients. Patients were divided into control (no-ACTH treatment group) and experimental (ACTH treatment group) groups according to whether patients received ACTH treatment for at least three cycles. Patients were excluded if they had normal serum cortisol levels (serum cortisol concentrations ≥85.3 nmol/L) or if their follow-up duration was <12 m.

### 2.2. ACTH treatment protocol

Patients in the ACTH treatment group were administered a short-acting formulation of animal-derived natural corticotropin (Shanghai the First Biochemical & Pharmaceutical Co Ltd, Shanghai, China) at a dosage of 0.4 IU/kg/day (total not exceeding 25U) intravenous continuously for five days for one treatment course, and one month separated each course (16).

### 2.3. Collection

A standardized data collection form was applied for all eligible patients. Data collected included: gender, age of onset, distribution of treating physicians, duration of the disease, pathological types, clinical type (1:SDNS, 2.FRNS, 3.SRNS), oral prednisolone dose (mg/kg) (T0: baseline, the time of the first ACTH application or discovered low serum cortisol concentration; T1: after 1 year; T2: currently), number of disease relapses, the time of first disease relapse, serum cortisol concentrations, the number of ACTH treatment courses (for ACTH treatment group only), evaluation of AEs, and prior or concomitant medications, including angiotensin-converting-enzyme inhibitors (ACEIs), immunosuppressive and cytotoxic drugs, and biological agents.

### 2.4. Data analysis

Statistical analysis was performed using SPSS Statistics v.26.0 (IBM Corp., Armonk, NY, United States). Normally and non-normally distributed continuous variables are presented as mean ± standard deviations (SD) or median (interquartile ranges, IQR) and were compared by independent sample *t*-test and Mann-Whitney *U* test, respectively. Categorical variables were compared by chi-square test or continuity corrected chi-square test or Fisher's exact test. The cumulative probability of relapse and time to first disease relapse was estimated according to Kaplan-Meier analysis. *p* < 0.05 was considered statistically significant.

## 3. Results

### 3.1. Study participants

A total of 56 NS patients who received ≥3 courses of ACTH treatment in our hospital from January 2018 to January 2021 were enrolled in the ACTH treatment group. Of these, five participants were excluded due to a serum cortisol concentration ≥85.3 nmol/L. Characteristics of patients in the ACTH group are presented in Supplementary Table S1. Cases included 51 patients across NS etiologies, including mesangial proliferative glomerulonephritis (MsPGN) (*n* = 17), IgM nephropathy (*n* = 5), focal segmental glomerulosclerosis (FSGS) (*n* = 6), minimal change disease (MCD) (*n* = 2), and 21 unbiopsied NS patients. The majority (40 of 51; 78.4%) of NS cases had failed ≥1 prior immunosuppressive or cytotoxic therapies, and 23 of 51 (45.1%) had failed ≥2 prior immunosuppressive and/or cytotoxic treatments other than oral corticosteroids. The most frequently used agents for NS treatment were tacrolimus (29 of 51, 56.9%), mycophenolate (23 of 51, 45.1%), and cyclosporin (7 of 51, 13.7%). Two patients had been treated with rituximab before being enrolled in the study. The ACTH group included 23 (45.1%) FRNS, 21 (41.2%) SDNS, and 7 (13.7%) SRNS patients.

Serum cortisol assays were performed in 48 NS patients that had not been treated with ACTH in our hospital from January 2018 to January 2021. Twenty-one of these patients had serum cortisol concentrations <85.3 nmol/L and were selected and enrolled in the control group. Characteristics of patients in the control group are presented in Supplementary Table S2. Renal pathologic types in the control group included MsPGN (*n* = 12), IgM nephropathy (*n* = 1), and unbiopsied (*n* = 8) NS patients. The majority (17 of 21, 81%) of patients had been treated with immunosuppressive agents other than oral corticosteroids but continued to experience disease relapses. Ten patients (10 of 21, 47.6%) had been treated with ≥2 agents before the start of this trial. The most frequently used agents were tacrolimus (16 of 21, 76.2%), mycophenolate (11 of 21, 52.4%), and cyclosporin (5 of 21, 23.8%). Three control patients had been treated with rituximab before being enrolled in the study. The no-ACTH control group included nine (42.9%) FRNS, 10 (47.6%) SDNS and two (9.5%) SRNS patients.

During the research, oral corticosteroids, and immunosuppressive and/or cytotoxic treatments were continued. Concurrent treatment with one or more immunosuppressive and/or cytotoxic treatments occurred in forty-seven (92.2%) and eighteen (85.7%) patients in the ACTH and control groups, respectively, throughout the study period. Concurrent treatment regiments with tacrolimus and prednisone were received by 18 of 51 (35.3%) patients, and mycophenolate and prednisone were received by 11 of 51 (21.6%) patients in the ACTH group. More than two immunosuppressive and/or cytotoxic treatments other than prednisone were received by 16 of 51 (31.4%) patients during ACTH treatment. In the control group, eight patients (8 of 21, 38.1%) continued to receive tacrolimus and prednisone and three patients (3 of 21, 14.3%) continued to receive mycophenolate and prednisone. Five patients (5 of 21, 23.8%) continued to receive more than two kinds of immunosuppressives other than prednisone during the study period in the control group. The most frequently used agents were tacrolimus, mycophenolate. In the ACTH group, twenty-nine (56.9%) patients received tacrolimus and the initiation mean dose of these 29 patients was 0.05 (0.03–0.07) mg/kg. In the control group, ten (47.6%) patients received tacrolimus, and the initiation mean dose of these 10 patients is 0.05 (0.04–0.06) mg/kg. Twenty (39.2%) patients received mycophenolate, and the initiation mean dose was 21.52 ± 6.11 mg/kg in the ACTH group. Seven (33.3%) patients received mycophenolate, and the initiation mean dose was 23.84 ± 4.00 mg/kg in the control group. Five patients and four patients were treated with rituximab in the ACTH group and control group respectively. No significant difference was found in these constituent ratios and mean doses (Table 1).

**Table 1:** Characteristics of patients.

Characteristics	ACTH treatment group, *n* = 51	No-ACTH Treatment Group, *n* = 21	Statistical Analyses, *p*
**Male, *n* (%)**	41 (80.4%)	19 (90.5%)	0.487
**Age at diagnosis, year, median (IQR)**	3 (2–6)	5 (3.0–5.5)	0.322
**Duration of disease at baseline, year, median (IQR)**	4 (1–6)	5 (3–7)	0.346
**Histopathology, *n* (%)**			0.263
MsPGN	17 (33.3%)	12 (57.1%)	
IgMN	5 (9.8%)	1 (4.8%)	
FSGS	6 (11.8%)	0 (0%)	
MCD	2 (3.9%)	0 (0%)	
Biopsy not performed	21 (41.2%)	8 (38.1%)	
**Clinical type, *n* (%)**			0.878
FRNS	23 (45.1%)	9 (42.9%)	
SDNS	21 (41.2%)	10 (47.6%)	
SRNS	7 (13.7%)	2 (9.5%)	
**Immunosuppressive regimens applied before enrolled, *n* (%)**			0.384
Only tacrolimus	8 (15.7%)	6 (28.6%)	
Only mycophenolate	9 (17.6%)	1 (4.8%)	
≥2 agents	23 (45.1%)	10 (47.6%)	
None	11 (21.6%)	4 (19.0%)	
**Immunosuppressive agent exposure before enrolled, *n* (%)**
Tacrolimus	29 (56.9%)	16 (76.2%)	0.124
Mycophenolate	23 (45.1%)	11 (52.4%)	0.574
Cyclosporin	7 (13.7%)	5 (23.8%)	0.487
Cyclophosphamide	5 (9.8%)	3 (14.3%)	0.891
Vincristine	5 (9.8%)	2 (3.9%)	1
Leflunomide	2 (3.9%)	0 (0%)	1
Rituximab	2 (3.9%)	3 (14.3%)	0.288
**Immunosuppressive regimens applied during study, *n* (%)**			0.591
Only tacrolimus	18 (35.3%)	8 (38.1%)	
Only mycophenolate	11 (21.6%)	3 (14.3%)	
Only cyclosporin	1 (2.0%)	2 (9.5%)	
Only Leflunomide	1 (2.0%)	0 (0.0%)	
None	4 (7.8%)	3 (14.3%)	
≥2 agents	16 (31.4%)	5 (23.8%)	
**Immunosuppressive agent exposure during study, *n* (%)**
Tacrolimus	29 (56.9%)	10 (47.6%)	0.474
Mycophenolate	20 (39.2%)	7 (33.3%)	0.639
Cyclosporin	1 (2.0%)	2 (9.5%)	0.202
Cyclophosphamide	2 (3.9%)	0 (0.0%)	1
Vincristine	5 (9.8%)	1 (4.8%)	0.815
Leflunomide	2 (3.9%)	0 (0.0%)	1
Rituximab	5 (9.8%)	4 (19.0%)	0.493

ACTH, adrenocorticotropic hormone; IQR, interquartile range; MsPGN, mesangial proliferative glomerulonephritis; IgMN, IgM nephropathy; FSGS, focal segmental glomerulosclerosis; MCD, minimal change disease; FRNS, frequent relapsing nephrotic syndrome; SDNS, steroid-dependent nephrotic syndrome; SRNS, steroid-resistant nephrotic syndrome.

### 3.2. Total group treatment response

**Figure 1:**
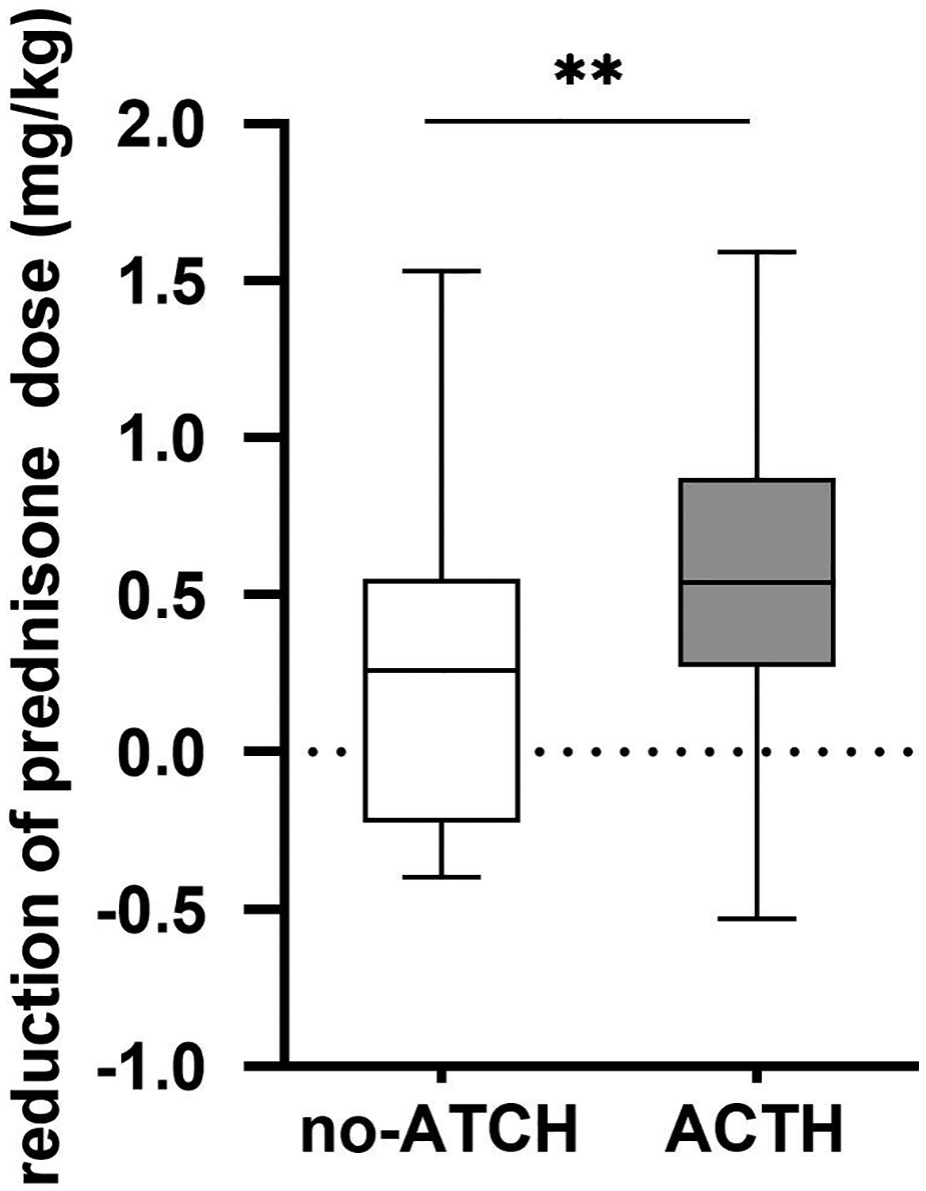
Reduction of prednisone maintenance dose 1 year after enrollment.

There was no significant difference in the sex distribution, distribution of treating physicians, age at diagnosis, or duration of disease and clinical type between the two groups (*p* > 0.05). The prednisone maintenance dose in the ATCH group at 1 year after receiving ACTH treatment was reduced by 0.603 ± 0.445 mg/kg. The prednisone maintenance dose in the control group was reduced by 0.267 ± 0.500 mg/kg at 1 year after enrollment in the study. The ACTH group presented a greater reduction in the prednisone maintenance dose compared with the control group (mean difference ± standard error of the mean (SEM) = 0.336 ± 0.1196 mg/kg, 95% confidence interval (CI) was 0.098 to 0.593, *p* = 0.006; Figure 1). After deleting the date of the patients who have received rituximab treatment during the study period, analysis based on the remaining date showed that the reduction of prednisone dose in the ACTH group remained greater than in the control group (mean difference ± SEM = 0.322 ± 0.136, 95%CI = 0.051 to 0.593, *p* = 0.021).

**Figure 2:**
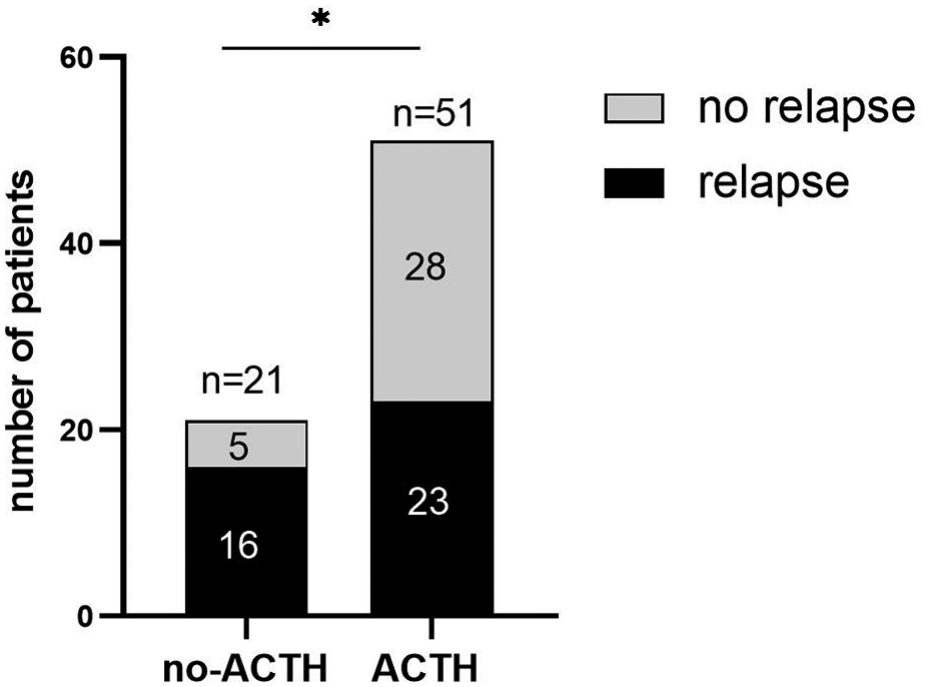
Bar diagram showing the proportion of relapses in each group.

**Figure 3:**
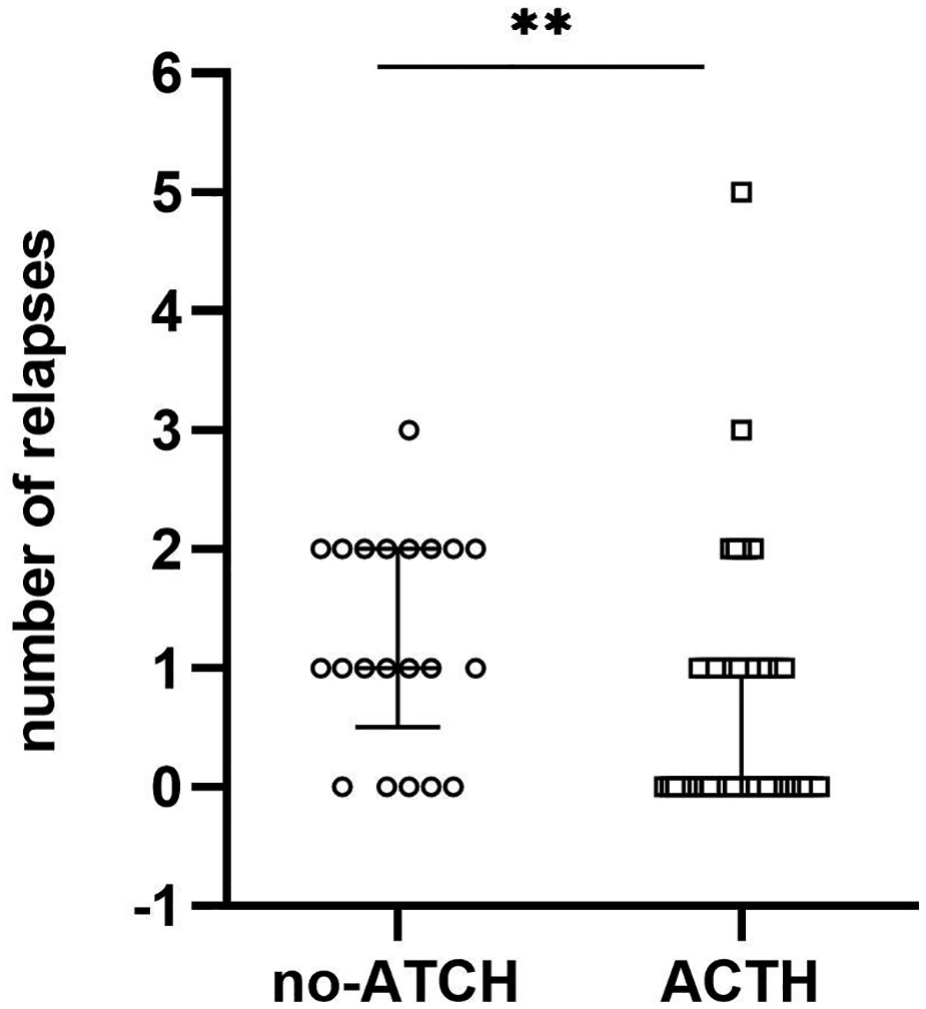
Numbers of recurrence in ACTH and no-ACTH control groups.

**Figure 4:**
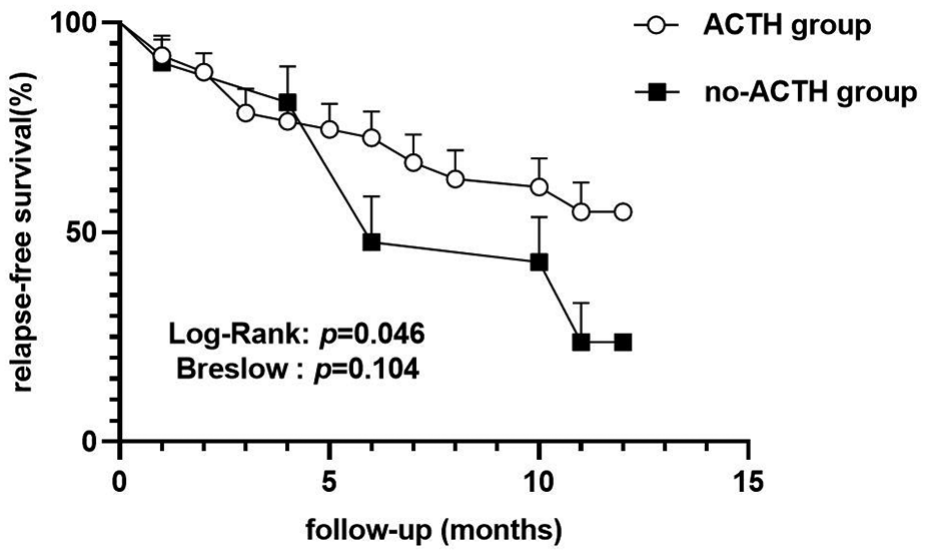
Kaplan-Meier plot of relapse-free survival.

In the ACTH group, 23 of 51 (45.1%) participants experienced ≥1 relapses within 12 m, with the median of the number of relapses being 0 (IQR = 0–1) during the 1-year follow-up period. In the control group, 16 of 21 (76.2%) participants experienced ≥1 relapses within 12 m, with the median of the number of relapses being 1 (IQR = 0.5–2). After 12 m of follow-up, the proportion of relapsed patients in the control group was higher than that in the ACTH group (odds ratio = 3.896, 95% CI = 0.093 to 0.843, *p* = 0.016; Figure 2) and the number of recurrences in the ACTH group was lower than that in the control group (median difference = −1, *p* = 0.006; Figure 3). Kaplan-Meier analysis was used to compare the time to first disease relapse between the two groups and the cumulative probability of relapse. Median survival time could not be estimated, as over 50% of the patients in ACTH group have no recurrence and were censored. The mean length of remission in the ACTH group was 8.902 m, and the mean time of relief in the control group was 7.905 m. Log-rank (Mantel-Cox) test showed *p* = 0.046, but Breslow test showed *p* = 0.104 (Figure 4). After deleted the data of the patients who have received rituximab treatment during the study period, the data for the remaining patients was analyzed by Chi-square test (*p* = 0.026) and the Mann-Whitney *U* test (*p* = 0.028) showed that the recurrence rate and the number of recurrences were still have a statistically significant difference between the two groups. However, Kaplan Meier analysis showed no significant difference between groups (log-rank test *p* = 0.067 and Breslow test *p* = 0.122).

### 3.3. Treatment response in the ACTH group

Fifty-one patients were treated with ACTH, receiving a mean of 6.2 ± 2.2 treatment courses (minimum and maximum were 3 and 13, respectively). Among the 51 patients, 17 (33.3%) patients received <6 courses of treatment, and 34 (66.7%) patients received ≥6 courses of treatment. Cortisol levels in 28 of 51(54.9%) patients returned to normal after treatment. The majority (20 of 28, 71.4%) of the patients in which cortisol levels returned to normal received ≥6 courses of treatment. Among the 28 patients whose cortisol levels returned to normal, an average of 4 courses of treatment (at least 1 treatment and at most 12 treatments) boosted cortisol levels to within the normal range. There was no difference in the prednisone maintenance dose reduction, the proportion of relapsed patients, the times of relapse, and time to first disease relapse between patients who received ACTH treatment <6 and ≥6 courses or between patients with and without normal cortisol levels (*p* > 0.05). Characteristics of patients in the ACTH treatment group are shown in Supplementary Table S2.

### 3.4. Extended follow-up

Thirteen patients were followed for more than 3 years, 27 patients were followed for 2–3 years, and 5 patients were followed for 1–2 years in the ACTH group. By February 2022, six patients were lost to follow-up after 1 year in the ACTH group. In the control group, two patients were followed for at least 3 years, 12 patients were followed for 2–3 years, and five patients were followed for 1–2 years. Two patients were lost to follow-up after 1 year in the control group. Ten patients in the ACTH group successfully discontinued all drug therapies, and no patients in the control group were able to discontinue any drug therapies. Among the 10 patients that discontinued drug treatment, 1 patient was weaned off all drugs during the first follow-up year and continued without drug intervention for 1.5 years.

### 3.5. Safety and tolerability

There were no serious AEs, although 13 patients reported AEs. The most common AEs was respiratory infections, with 13.7% (7 of 51) of patients reporting a respiratory infection; however, none of the respiratory infections were deemed related to the study. Ocular hypertension was observed in 3.9% (2 of 51) of patients and corrected with brinzolamide eye drops. Hypertension was observed in 2.0% (1 of 51) of patients and was treated with amlodipine. Three patients developed anaphylaxis in the 5th, 8th and 9th course of ACTH treatment respectively.

## 4. Discussion

This is the largest retrospective, observational comparative study published to date that examines the efficacy and safety of ACTH in the treatment of childhood patients with NS. Overall, the decrease of prednisone maintenance dose in the ACTH group was more significant than that in the no-ACTH control group at 12 months (mean difference ± SEM = 0.336 ± 0.1196 mg/kg). In addition, ACTH treatment resulted in fewer numbers of disease relapse (median = 0, IQR = 0–1) compared to the control group (median = 1, IQR = 0.5–2) and a lower disease recurrence rate (45.1%) compared to the control group (76.2%).

About 40% of children with NS will experience FRNS or develop SDNS (15). The incidence of SRNS is gradually increasing, accounting for nearly 15%–30% of all NS cases (17, 18). These patients require treatment with agents other than glucocorticoids, including immunosuppressants and biological agents. However, a subset of these patients also fail these complementary therapies and thus urgently need evaluation of additional treatment options. ACTH, a 39 amino acid straight chain polypeptide, plays a pivotal role in the hypothalamic–pituitary-adrenal axis and is important in maintaining homeostasis in the neuroimmune endocrine system. It is secreted by the pituitary gland, inhibited by adrenocortical hormone, and regulated by corticotropin-releasing hormone (19–21). ACTH acts as an agonist of the melanocortin receptors (MCRs) in addition to its role in regulating adrenocortical hormones (8, 21, 22). ACTH protects the kidney *via* both steroidal and nonsteroidal pathways by activating different MCRs (8, 21). ACTH is able to bind all five MCRs. MCRs are expressed in a variety of cells and play an active role in modulation of exocrine function, lipolysis, and anti-inflammation (19). Guan X et al. found that ACTH exerts protective effects in nephrotoxic serum (NTS)-injury wild-type mice, and the protective efficacy of ACTH was blunted in NTS-injured MC1R^e/e^ mice (e/e mice; homozygous for the mutant recessive *Mc1r^e^* allele). Another study in 2022 also suggested that MC1R knockout worsened passive Heymann nephritis. These results suggest that MC1R signaling may plays a key role in the kidney protective effects of ACTH (23, 24). Furthermore, it has been shown that NTS-injured wild type mice treated with MS05 (a selective MC1R agonist) have similar results as those treated with ACTH, but to a lesser extent (24). Strong expression of MC1R and MC5R and weak expression of MC2R and MC4R have been detected in the human kidney (8, 25). These studies suggest that not only MC1R but also other MCRs may play a role in protecting the kidney. In addition, renal protection may occur *via* neurogenic anti-inflammatory effects and secondary to dyslipidemia correction and increased plasma insulin levels that are mediated by ACTH (8, 21, 25, 26).

ACTH treatment for NS has gained popularity over the last decade, following a study that showed that a synthetic ACTH analogue (ACTH 1–24; Synacthen Depot) reduced proteinuria in patients with differing pathologic changes (27). More recently, several case series of patients with immunosuppressive and steroid-resistant NS suggest that ACTH has effects beyond steroidogenesis and indicate that ACTH may be used in the treatment of FRNS, SDNS, and SRNS (9, 19, 21). Most previous studies have reported changes in the degree of proteinuria in NS patients treated with ACTH when comparing post-treatment levels to pretreatment levels (9, 19, 21, 28–30). The main outcome of interest in our study was to determine the ability of ACTH to reduce the glucocorticoid hormone dosage and prevent disease relapse. Here, the mean maintenance dose of prednisone was reduced at 1 year in patients that received ACTH treatment, and the extent of this reduction in the ACTH group was greater than in the no-ACTH control group. This is in accordance with a retrospective review conducted by Han et al. that investigated the effects of ACTH in treating childhood FRNS, though there was no control group in this previous study (31). Several studies have shown unfavorable results with ACTH treatment. For example, van de Logt et al. showed that synthetic ACTH is less effective than cyclophosphamide in inducing NS remission in high-risk patients with idiopathic membranous nephropathy (32). In 2018, Wang C et al. conducted a randomized control trial to investigate the ability of ACTH to prevent disease relapse in FRNS and SDNS patients, with outcomes assessed within the first six months of treatment; however, the trial was terminated prematurely for lack of discernible treatment efficacy (5). In the present study, the proportion of relapsed patients in the ACTH group was lower than that in the no-ACTH control group and the median of the number of disease recurrences in the ACTH group was less than in the control group. Furthermore, the mean length of remission in the ACTH group is longer than that in the control group. Although the Fisher's exact test and Mann-Whitney *U* test showed that there was a statistically significant difference between the two groups in the proportion of relapses and frequency of recurrence, the results of the two statistical testing methods of the Kaplan Meier analysis were inconsistent (log-rank test showed *p* = 0.046 and Breslow test showed *p* = 0.104). The log-rank test is sensitive to long-term differences and Breslow tests give greater weight to recent differences in outcome events. Thus, it is possible that there was no difference at the beginning of the study period between two groups, although a difference in maintaining remission between the two groups appeared over time. After deleted the data of the patients who have received rituximab treatment during the study period, Kaplan Meier analysis based on the remaining data showed no statistically significant differences between groups. This result may be due to the small number of included cases or the additive effect of several medications. By February 2022, 10 patients in the ACTH group and no patients in the control group had successfully discontinued all drug therapies. Thus, adjuvant ACTH may prevent disease relapses. The present study results are inconsistent with Wang C et al.'s study. Differences between the studies may be due to the fact that the majority of patients in the current study were concurrently treated with ≥1 immunosuppressive agents during the study period, whereas patients in the Wang C et al.'s study stopped other relapse-preventing treatments, including corticosteroids and other immunosuppressive therapies, after a two-week overlap with ACTH. Two IgA nephropathy patients exhibited complete remission when administered ACTH combination therapy with cyclophosphamide in the report of Prasad et al. (13). Combination therapy with acthar gel and tacrolimus significantly reduced proteinuria and improved clinical response rates in patients with treatment-resistant MGN and FSGS compared with acthar gel alone (14). Thus, the combination of ACTH and immunosuppressive agents that simultaneously targets different pathogenic pathways may confer superior benefits to NS patients.

In the ACTH group, cortisol levels in 28 of 51 (54.9%) patients were normal after treatment. The majority (20 out 28, 71.4%) of the patients that recovered normal cortisol levels received ≥6 treatments. There was no statistical difference in the reduction of the mean maintenance dose of prednisone and disease relapse between the patients with and without normal cortisol levels. This result may be due to ACTH exerts cortisol-independent, renal-protecting activity. In the ACTH group, ≥6 courses of ACTH treatment were received by 34 patients. However, there was no statistical difference in the reduction in the mean maintenance dose of prednisone and disease relapse between the patients that had been treated <6 courses and those that had been treated ≥6 course. These results differ from previous research, which has shown that proteinuria is inversely related to the cumulative ACTH dose (33). The current study does not support a cumulative dose effect in the efficacy of ACTH treatment in childhood NS patients. Shrivastava et al. reported a patient with steroid-dependent FSGS who developed significant remission in proteinuria when given repository porcine ACTH treatment; however, following treatment the proteinuria and anasarca re-appeared, and high titers of *de novo* IgG antibodies that were reactive to the porcine corticotropin were found in the patient's serum (11). Pei Wang et al. also reported *de novo* formation of IgM and IgG antibodies in an adolescent SRNS patient's serum that were reactive to corticotropin following animal-derived natural corticotropin treatment, indicating an acquired resistance to corticotropin therapy (12). In the present study, the possibility that neutralizing antibodies formed and caused a relapse after remission of NS and affect the cumulative dose effect cannot be ruled out.

According to current reports, the side effects of ACTH are tolerable and include the following: increased swelling, obesity, hypertension, hyperglycemia, osteoporosis, upper respiratory infection, mood changes, sleep disorders, behavior disorders, and skin disease (10, 21). Because of its infrequent use, the side effects of ACTH are not well documented in pediatric NS patients. In the present study, ACTH was also generally well-tolerated with few patients experiencing mild side effects.

There have been few case-controlled studies on pediatric NS and ACTH treatment in the past (34). The present study was a comparative study performed to determine the efficacy and safety of ACTH in treating pediatric patients with NS of varying etiologies. There are several limitations in this study. The study is a retrospective clinical study that includes patients receiving various combination treatments. This limits our ability to definitively implicate ACTH as the reason for improvement. It is unknown whether the observed treatment effectiveness was the result of a true additive effect between immunosuppressive agents and ACTH or simply a reflection of ACTH therapy.

## 5. Conclusion

In summary, this retrospective comparative study found that the addition of ACTH therapy to conventional therapy seems to be a potential therapeutic modality for patients with FRNS, SDNS, and SRNS, and that ACTH was well-tolerated without any major AEs. Additionally, large-scale prospective, randomized controlled studies are needed to assess the efficacy of ACTH in childhood refractory NS.

## Data Availability

The original contributions presented in the study are included in the article/**Supplementary Material**, further inquiries can be directed to the corresponding author/s.
