# Supplementary material for: Real-word adrenocorticotropic hormone treatment for childhood-onset nephrotic syndrome
Source: Front Pediatr. 2023 Mar 9;11:1044075. doi: 10.3389/fped.2023.1044075 (PMC10036038; doi:10.3389/fped.2023.1044075)
Supplement: Supplementary file 1 [file Table1.docx]

**Supplementary table 1.** Characteristics of patients in the ACTH treatment group.

| Patient | Age of onset  (year) | Disease  course  (year) | Renal pathology | Clinical type | Number of   treatment courses | Reduction of prednisone  (mg/kg) | The number of disease recurrences | Time to first disease relapse (month) | Previous IST/BT | Concurrent IST/BT |
| --- | --- | --- | --- | --- | --- | --- | --- | --- | --- | --- |
| 1 | 3 | 4 | MsPGN | FRNS | 5 | 0.40 | 2 | 2 | CTX, TAC, VCR | TAC, VCR |
| 2 | 1 | 8 | MsPGN | SDNS | 6 | 0.00 | 1 | 11 | CSA, MMF, TAC,  rituximab | MMF,CTX, LEF |
| 3 | 10 | 4 | MsPGN | SDNS | 6 | 0.41 | 0 | NA | MMF | MMF |
| 4 | 2 | 3 | IgMN | FRNS | 7 | 0.18 | 1 | 8 | MMF, TAC | TAC, rituximab |
| 5 | 5 | 2 | MsPGN | FRNS | 11 | 0.50 | 1 | 2 | TAC, MMF | MMF |
| 6 | 7 | 5 | FSGS | SRNS | 7 | 0.65 | 0 | NA | CTX, TAC, VCR | TAC |
| 7 | 2 | 6 | NA | FRNS | 7 | 0.57 | 0 | NA | CSA, LEF | LEF |
| 8 | 2 | 1 | NA | FRNS | 3 | 0.00 | 1 | 11 | MMF, TAC | TAC |
| 9 | 7 | 1 | MsPGN | SDNS | 8 | 1.42 | 0 | NA | NONE | NONE |
| 10 | 4 | 5 | MsPGN | FRNS | 9 | 0.56 | 1 | 3 | TAC | TAC |
| 11 | 1 | 4 | MsPGN | SRNS | 8 | 1.59 | 0 | NA | TAC | MMF |
| 12 | 11 | 1 | MsPGN | FRNS | 7 | 0.29 | 0 | NA | MMF | MMF, TAC |
| 13 | 2 | 0.3 | NA | SDNS | 5 | 1.07 | 0 | NA | NONE | TAC |
| 14 | 2 | 10 | FSGS | SRNS | 5 | 0.65 | 0 | NA | MMF, TAC | TAC |
| 15 | 2 | 13 | NA | SDNS | 6 | 0.27 | 0 | NA | MMF | MMF |
| 16 | 3 | 9 | FSGS | FRNS | 13 | 0.35 | 0 | NA | MMF, TAC | TAC, MMF |
| 17 | 9 | 4 | MsPGN | FRNS | 5 | 0.45 | 0 | NA | TAC, VCR | TAC |
| 18 | 3 | 8 | IgMN | SRNS | 10 | 0.87 | 0 | NA | MMF, TAC, CTX | CTX, MMF |
| 19 | 3 | 6 | NA | SDNS | 7 | 0.22 | 0 | NA | MMF | MMF |
| 20 | 3 | 2 | NA | FRNS | 8 | 1.17 | 0 | NA | NONE | NONE |
| 21 | 5 | 8 | MsPGN | FRNS | 7 | -0.53 | 1 | 11 | MMF | NONE |
| 22 | 3 | 5 | NA | SDNS | 6 | 1.03 | 0 | NA | MMF | MMF |
| 23 | 2 | 8 | NA | SDNS | 6 | 0.22 | 1 | 7 | CSA, TAC, MMF | TAC |
| 24 | 4 | 1 | NA | SDNS | 6 | 1.05 | 0 | NA | NONE | MMF |
| 25 | 5 | 1 | NA | SDNS | 6 | 1.14 | 0 | NA | NONE | NONE |
| 26 | 2 | 4 | FSGS | SRNS | 6 | 0.31 | 5 | 3 | TAC, VCR | TAC,VCR |
| 27 | 3 | 6 | MsPGN | SDNS | 8 | 0.54 | 1 | 8 | MMF, | MMF |
| 28 | 3 | 10 | MsPGN | SDNS | 8 | 0.11 | 2 | 1 | CSA, TAC | TAC, VCR |
| 29 | 9 | 5 | FSGS | SDNS | 3 | 0.18 | 1 | 1 | TAC, LEF, CTX | MMF, rituximab |
| 30 | 3 | 4 | NA | SDNS | 4 | 0.71 | 1 | 3 | NONE | CSA |
| 31 | 2 | 5 | MsPGN | SDNS | 6 | 1.25 | 0 | NA | TAC | TAC,VCR |
| 32 | 4 | 4 | NA | SDNS | 5 | 0.52 | 0 | NA | MMF | TAC |
| 33 | 6 | 3 | NA | FRNS | 3 | 0.50 | 1 | 3 | TAC | TAC, rituximab |
| 34 | 4 | 4 | MsPGN | SDNS | 6 | 0.13 | 2 | 1 | TAC, MMF | MMF, TAC |
| 35 | 7 | 5 | NA | SDNS | 5 | 0.41 | 0 | NA | MMF, TAC | TAC |
| 36 | 2 | 6 | NA | SRNS | 3 | 0.45 | 0 | NA | CSA, TAC | TAC |
| 37 | 1 | 1 | NA | SRNS | 7 | 1.54 | 0 | NA | TAC | TAC |
| 38 | 5 | 3 | NA | FRNS | 6 | 0.65 | 0 | NA | NONE | TAC |
| 39 | 13 | 1 | IgMN | FRNS | 8 | 0.79 | 0 | NA | MMF | TAC |
| 40 | 2 | 0.6 | NA | FRNS | 7 | 1.24 | 0 | NA | NONE | MMF |
| 41 | 3 | 0.4 | MCD | FRNS | 9 | 1.38 | 0 | NA | TAC | TAC |
| 42 | 8 | 6 | MsPGN | SDNS | 3 | 0.69 | 0 | NA | MMF, TAC | TAC |
| 43 | 12 | 1 | IgMN | FRNS | 7 | 0.66 | 0 | NA | NONE | MMF |
| 44 | 3 | 1 | NA | SDNS | 3 | 0.83 | 2 | 6 | NONE | MMF |
| 45 | 2 | 8 | MsPGN | FRNS | 6 | 0.25 | 1 | 5 | MMF, CSA, TAC, CTX | MMF, rituximab |
| 46 | 5 | 8 | NA | SDNS | 9 | 0.11 | 3 | 4 | CSA, MMF | MMF, VCR |
| 47 | 7 | 0.8 | MsPGN | FRNS | 5 | 1.13 | 2 | 1 | NONE | MMF, TAC |
| 48 | 3 | 9 | FSGS | FRNS | 6 | 0.31 | 1 | 7 | TAC, VCR, rituximab | TAC, rituximab |
| 49 | 10 | 4 | MCD | FRNS | 4 | 0.65 | 1 | 7 | TAC | TAC |
| 50 | 3 | 9 | NA | FRNS | 3 | 0.25 | 1 | 10 | TAC | TAC |
| 51 | 2 | 4 | IgMN | FRNS | 3 | 0.64 | 1 | 3 | MMF, TAC | TAC |

Abbreviations: IST immunosuppressive therapy, BT biological therapy, VCR vincristine, TAC tacrolimus, LEF leflunomide, MMF mycophenolate mofetil, CSA cyclosporin, CTX cyclophosphamide, NA not available, MsPGN mesangial proliferative glomerulonephritis, IgMN IgM nephropathy, FSGS focal segmental glomerulosclerosis, MCD minimal change disease, SDNS steroid-dependent nephrotic syndrome, FRNS frequent relapses NS nephrotic syndrome, SRNS steroid-resistant NS nephrotic syndrome.

**Supplementary table 2**. Characteristics of patients in the no-ACTH control group.

| Patient | Age of onset  (year) | Disease  course  (year) | Renal pathology | Clinical  type | Reduction of prednisone  (mg/kg) | The number of disease recurrences | Time to first disease relapse (month) | Previous IST/BT | Concurrent IST/BT |
| --- | --- | --- | --- | --- | --- | --- | --- | --- | --- |
| 1 | 1 | 8 | MsPGN | FRNS | -0.19 | 3 | 1 | CSA, TAC, MMF | TAC, rituximab |
| 2 | 5 | 2 | NA | SDNS | 0.36 | 0 | NA | NONE | NONE |
| 3 | 9 | 2 | NA | SDNS | 0.91 | 0 | NA | TAC | TAC |
| 4 | 6 | 5 | MsPGN | SDNS | 0.35 | 2 | 6 | TAC | NONE |
| 5 | 5 | 6 | MsPGN | SDNS | 0.85 | 0 | NA | NONE | NONE |
| 6 | 8 | 2 | MsPGN | SDNS | 0.83 | 0 | NA | MMF, TAC | TAC |
| 7 | 11 | 4 | MsPGN | FRNS | 0.33 | 2 | 1 | TAC, MMF | TAC, VCR, MMF |
| 8 | 1 | 8 | IgMN | SRNS | 0.26 | 1 | 11 | TAC, CTX, rituximab, MMF | MMF, rituximab |
| 9 | 3 | 1 | MsPGN | FRNS | 1.53 | 0 | NA | TAC | TAC |
| 10 | 8 | 3 | NA | SDNS | 0.74 | 1 | 10 | TAC | TAC |
| 11 | 4 | 6 | NA | FRNS | -0.33 | 1 | 11 | MMF | MMF |
| 12 | 3 | 7 | MsPGN | FRNS | 0.25 | 1 | 11 | MMF, TAC, CTX, CSA | CSA |
| 13 | 4 | 5 | NA | SDNS | -0.4 | 2 | 6 | TAC | TAC |
| 14 | 5 | 10 | MsPGN | FRNS | 0.08 | 2 | 4 | CSA, TAC, VCR, MMF, rituximab | MMF, rituximab |
| 15 | 5 | 5 | MsPGN | SDNS | -0.26 | 2 | 6 | NONE | MMF |
| 16 | 2 | 3 | MsPGN | FRNS | 0.31 | 1 | 6 | MMF, TAC | TAC |
| 17 | 3 | 7 | NA | SRNS | 0.25 | 1 | 11 | MMF, TAC, CTX, CSA | CSA |
| 18 | 4 | 5 | NA | SDNS | -0.4 | 2 | 6 | TAC | TAC |
| 19 | 5 | 10 | MsPGN | FRNS | 0.08 | 2 | 4 | CSA, TAC, VCR, MMF, rituximab | MMF, rituximab |
| 20 | 5 | 5 | MsPGN | SDNS | -0.26 | 2 | 6 | NONE | MMF |
| 21 | 2 | 3 | NA | FRNS | 0.31 | 1 | 6 | MMF, TAC | TAC |
